# Retraction Note: Daylight saving time affects European mortality patterns

**DOI:** 10.1038/s41467-026-73429-x

**Published:** 2026-05-28

**Authors:** Laurent Lévy, Jean-Marie Robine, Grégoire Rey, Raúl Fernando Méndez Turrubiates, Marcos Quijal-Zamorano, Hicham Achebak, Joan Ballester, Xavier Rodó, François R. Herrmann

**Affiliations:** 1 Medical School of the University of Geneva, Geneva, Switzerland; 2 Institut National de la Santé et de la Recherche Médicale (INSERM), Montpellier, France; 3 École Pratique des Hautes Études, Paris, France; 4 CépiDc-Inserm, Hôpital Bicêtre, Paris, France; 5 ISGlobal, Barcelona, Spain; 6 Universitat Pompeu Fabra (UPF), Barcelona, Spain; 7 Institució Catalana de Recerca i Estudis Avançats, Barcelona, Catalonia Spain; 8 Division of Geriatrics, Department of Rehabilitation and Geriatrics, Geneva University Hospitals, Thônex, Switzerland

Retraction note to: *Nature Communications* 10.1038/s41467-022-34704-9, published online 14 November 2022

The Editors have retracted this article.

After publication, the Editors were informed that the statistical model presented in this article could be inaccurate and that, therefore, the article’s main finding, “that mortality decreases in spring and increases in fall during the first two weeks following each DST [daylight saving time] transition” was not adequately substantiated. These criticisms, originally raised by Klerman, E.B., Weaver, M.D., Roenneberg, T. et al., were published as a Matters Arising, ^1^.

The authors provided *Nature Communications* with a reply ^2^ to ^1^, together with additional statistical data, which were assessed internally by the editors alongside input from an external reviewer. When analyses were repeated using restricted time windows around DST transitions, the associations reported in the original article were not replicated.

The Editors, therefore, consider that the main finding of this article remains unconfirmed.

The authors disagree with this retraction. The Journal has been informed that Francois Hermann has passed away.
